# Acute Caffeinated Coffee Consumption Does not Improve Time Trial Performance in an 800-m Run: A Randomized, Double-Blind, Crossover, Placebo-Controlled Study

**DOI:** 10.3390/nu10060657

**Published:** 2018-05-23

**Authors:** Alexandre C. Marques, Alison A. Jesus, Bruna M. Giglio, Ana C. Marini, Patrícia C. B. Lobo, João F. Mota, Gustavo D. Pimentel

**Affiliations:** Clinical and Sports Nutrition Research Laboratory (Labince), Faculty of Nutrition, Federal University of Goias, Goiânia, GO 74605-080, Brazil; alexandre.c.marques@hotmail.com (A.C.M.); alison_deus@hotmail.com (A.A.J.); brunamgiglio@gmail.com (B.M.G.); ac.marini22@gmail.com (A.C.M.); patriciacristina.nutri@gmail.com (P.C.B.L.); jfemota@gmail.com (J.F.M.)

**Keywords:** coffee, caffeine, run, performance, time trial

## Abstract

Introduction: Studies evaluating caffeinated coffee (CAF) can reveal ergogenic effects; however, studies on the effects of caffeinated coffee on running are scarce and controversial. Aim: To investigate the effects of CAF consumption compared to decaffeinated coffee (DEC) consumption on time trial performances in an 800-m run in overnight-fasting runners. Methods: A randomly counterbalanced, double-blind, crossover, placebo-controlled study was conducted with 12 healthy adult males with experience in amateur endurance running. Participants conducted two trials on two different occasions, one day with either CAF or DEC, with a one-week washout. After arriving at the data collection site, participants consumed the soluble CAF (5.5 mg/kg of caffeine) or DEC and after 60 min the run was started. Before and after the 800-m race, blood pressure and lactate and glucose concentrations were measured. At the end of the run, the ratings of perceived exertion (RPE) scale was applied. Results: The runners were light consumers of habitual caffeine, with an average ingestion of 91.3 mg (range 6–420 mg/day). Time trial performances did not change between trials (DEF: 2.38 + 0.10 vs. CAF: 2.39 + 0.09 min, *p* = 0.336), nor did the RPE (DEC: 16.5 + 2.68 vs. CAF: 17.0 + 2.66, *p* = 0.326). No difference between the trials was observed for glucose and lactate concentrations, or for systolic and diastolic blood pressure levels. Conclusion: CAF consumption failed to enhance the time trial performance of an 800-m run in overnight-fasting runners, when compared with DEC ingestion. In addition, no change was found in RPE, blood pressure levels, or blood glucose and lactate concentrations between the two trials.

## 1. Introduction

Caffeine has been widely used as an ergogenic aid to increase physical performance [1,2]. It is available in different forms, such as gels, gums, powders, bars, and energy drinks [1]; however, few studies have investigated the effects of caffeinated coffee (CAF) on runners [3,4,5].

Clarke et al. [3] investigated the effects of CAF (containing 3 mg/kg of b.w.), decaffeinated coffee (DEC), and a placebo after one hour of rest and found that CAF ingestion led to a 1.3% and 1.9% faster performance in a race when compared to DEC or a placebo (water), respectively. In addition, Wiles et al. [5] observed that consumption of 3 g (150–300 mg/caffeine) of CAF an hour before a treadmill test was able to improve the performance (time to run 1500 m) by 1.45% when compared to DEC. On the other hand, Graham et al. [4] also evaluated the consumption of CAF (containing 4.5 mg/kg of b.w.) one hour before a treadmill exercise and did not find a difference between CAF, DEC or a placebo (dextrose) in endurance performance times. Therefore, the contradictory findings on running performance may be linked to variations in coffees nutritional properties, such as the chlorogenic acid and caffeine content [6], differences in coffee-derived caffeine content due to changes in weather, coffee processing (roasting, processing, storage), and genetic engineering [7]. In addition, a fed or fasted state [8] and the placebo effect [9,10] may be responsible for variations in performance. Recent evidence suggests that inter-individual variations in polymorphisms of CYP1A2 and ADORA2A lead to different responses on run performance. The first gene is responsible for metabolizing the caffeine in paraxanthine, theobromine, and theophylline; the second gene regulates the adenosine receptors [11]. No less important, differences in run locale, track type, and equipment can impair the performance.

Several studies have demonstrated that caffeine has ergogenic effects, due to central nervous system inhibition of adenosine receptors [12], enhanced adrenaline secretion [4], and reduced ratings of perceived exertion (RPE) [13]. Although it has already been observed that acute supplementation of caffeine (5 mg/kg of b.w.) one hour prior to exercise may improve performance in high-intensity cycling with low carbohydrate availability through enhanced anaerobic contribution [8], the effects of caffeine powder ingestion on a time trial performance in low carbohydrate availability conditions have not been measured before. In addition, evidence suggests that caffeine supplementation (5 mg/kg of b.w.) may increase performance, as well as anaerobic energy sources, in high-intensity running [14].

Coffee is one of the most consumed beverages in the world. Caffeine can provide ergogenic effects and increase anaerobic energy provision. However, beverages with coffee resemble more with the habitual of the practitioners of exercises than caffeine capsules. We hypothesize that CAF ingestion 60 min prior to an 800-m run, when compared to DEC consumption, could improve time trial performance in overnight-fasting runners. Therefore, we sought to investigate the effects of CAF consumption compared to DEC on time trial performance in an 800-m run in overnight-fasting runners.

## 2. Materials and Methods

### 2.1. Design Study and Participants

A randomly counterbalanced, double-blind, crossover, placebo-controlled study was conducted with 12 healthy adult males with experience in amateur endurance running and a mean age of 23.50 ± 3.94 years. The trials were conducted on a 400-m athletic track. The sample size was determined based on a pilot study from our group, 10 subjects were necessary to detect a statistical difference. We used a margin of error of 5% and a confidence level of 95%. All subjects gave their informed consent for inclusion before they participated in the study. The study was conducted in accordance with the Declaration of Helsinki and the protocol was approved by the Ethics Committee of Federal University of Goiás (2.361.759 version 2).

### 2.2. Habitual Food Intake Recording and Caffeine-Containing Foods

Two days before the test, the runners were instructed to maintain their food standard and hydration. The consumption of calories, carbohydrates, total proteins, and total lipids were determined using habitual 24-h dietary recall. The TACO^®^ database was used to quantify macronutrient intake and DietBox^®^ software for nutrient calculation. To evaluate food frequency and caffeine quantity of foods, we used a questionnaire adapted from a validated instrument [15] and applied by trained nutritionists. Caffeine content was obtained from the USDA Food Composition Databases or from food labels.

### 2.3. Coffee Preparation

The runners were instructed not to consume caffeinated beverages within the 48 h before trials to isolate the acute effects of possible food-derived caffeine ingestion. Indeed, caffeine’s ergogenic aid did not affect the withdrawal of food sources or supplementation of caffeine [16]. The overnight fasting occurred 12 h prior to trials to simulate low carbohydrate availability conditions among runners.

Participants conducted two trials on two different occasions, one day with either CAF or DEC, with a one-week interval between trials (washout period). After overnight fasting of 8–10 h, all runners were driven to the athletics track (local data collection) and consumed soluble CAF (5.5 mg/kg caffeine) or DEC (Nescafé^®^, Nestlé, Brazil) from identical batches.

The caffeine content of soluble coffee was obtained from a previous study [17], which has, on average, 3.4 g of raw caffeine in 100 g of coffee. An external investigator measured the amount of soluble coffee to obtain 5.5 mg/kg of caffeine. Therefore, to obtain this amount of caffeine per runner, we needed to weigh 12.8 ± 1.3 g/runner of soluble CAF and DEC, which resulted in 378.5 ± 40.2 mg of caffeine. The same quantity of DEC was weighted.

Two trials (CAF and DEC) were dissolved in 200 mL of hot water (approximately 60 °C) and served in plastic cups. Both experiments were dissolved in a cup containing 200 mL of water.

Runners had two minutes to ingest the cup of coffee; when it was completely finished, the time was counted up to 60 min to reach the maximum concentration of caffeine in their blood. Both beverages (CAF and DEC) were prepared identically in amount, appearance, and water temperature. They were prepared and separated by external investigators to ensure the placebo and double-blind effects.

### 2.4. Data Collection

Anthropometric measures, such as body weight, height, and skinfolds, were collected before characterization tests. Blood pressure, lactate, and glucose were determined during the fasting and immediately after the running test. To obtain body weight, a balance scale was used; for height measurement, a stadiometer was used to calculate the body mass index (BMI), using the body weight/height^2^ formula. For characterization of adiposity, skinfold measurements such as triceps, biciptal, subscapular, and suprailiac were performed before the trial using a Lange^®^ adipometer.

Waist circumference was assessed using inelastic tape and measurements were obtained at the iliac crest and midpoint between the last ribs. Thigh circumference was measured at the mid-point of the thigh, between the proximal border of the patella and inguinal fold. Calf circumference was assessed in a plane perpendicular to the calf (for the most part), with the subject sitting on a chair with his right foot resting on the floor.

Systolic and diastolic blood pressure levels were obtained before and immediately after running by means of GTECH^®^ equipment. Blood lactate and glycaemia concentrations were obtained from peripheral blood collected by a lancet puncture before and after running, using a portable lactate analyzer (Accutrend Lactate, Roche Diagnostics, Mannheim, Germany). A GTECH^®^ analyzer was used to determine the glycaemia concentrations. After the race, the same initial data were collected.

### 2.5. Race Procedures

A week prior to the test, a familiarization running session was performed. The two trial applications were random; after overnight fasting of 8–10 h and then ingesting coffee and waiting for 60 min, immediately after they were positioned on the athletics track. The trials began with the following instructions: “On your marks”, “get set”, and “go”. To complete the 800-m race, runners had to finish two laps of the 400-m athletics track.

Participants were encouraged to perform the race with maximum effort, receiving motivating words like “let’s go” and “running out”. After completing the race, the RPE on the Borg scale [18] was determined, and then glucose, lactate, and blood pressure collection was performed again. Before running, the runners maintained their normal hydration; during the race no runner ingested water due to the short duration of the run.

### 2.6. Statistical Analyses

Statistical analyses were described by mean and standard deviation. The ANOVA test was applied to verify possible differences in glucose and lactate concentrations and systolic and diastolic blood pressure. In addition, t-test was performed to verify possible differences in performance (time trial) and RPE. An outlier was removed just of time trial analysis. A *p* < 0.05 was considered statistically significant. Medcalc^®^ software (MedCalc, Mariakerke, Belgium) was used for all analyses.

## 3. Results

### 3.1. General Characteristics of Runners

Both trials were performed in the morning, with an average temperature of 21.38 ± 0.49 °C and a relative air humidity of 88.33 ± 3.76%. In addition, no difference was detected in temperature (21.33 ± 0.49 vs. 21.42 ± 0.51, *p* > 0.05) or humidity (89.00 ± 3.36 vs. 87.67 ± 4.16, *p* > 0.05) in the DEC vs. CAF group (Table 1).

According to BMI, all runners were eutrophic and presented adiposity, evaluated by waist circumference, within the normal classification (Table 1). The average training was 4.92 (range of 2–7) times per week and a running distance of 2.8 km (range 1–9) per training session (Table 1).

### 3.2. Habitual Food and Caffeine Consumption

**Figure 1:**
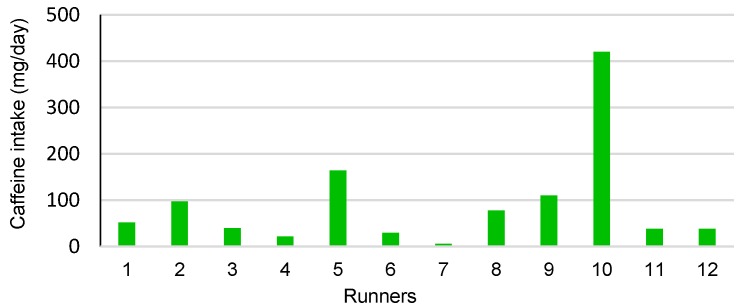
Habitual caffeine intake (mg/day) among runners.

Table 2 shows the food intake. The runners’ habitual diet was adequate in carbohydrates (4.0 g/kg of b.w.), protein (1.9 g/kg of b.w.), and lipids (1.0 g/kg of b.w.) [19]. Table 3 shows the frequency and quantity of food sources of caffeine. Food sources of caffeine were CAF, caffeine powder, a cola soft drink, chocolate, and a ready-to-drink chocolate beverage. Runners were light consumers of caffeine, with an average ingestion of 91.3 mg (range 6–420) per day (Table 3). Figure 1 shows the habitual caffeine consumption per runner, with a range of 6–420 mg/day.

### 3.3. Glucose and Lactate Concentrations

**Figure 2:**
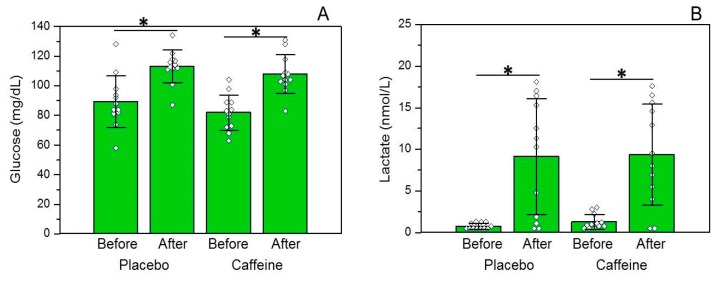
Blood glucose and lactate concentrations before and after the trials. (**A**) blood glucose concentrations (*n* = 12) and (**B**) blood lactate concentrations (*n* = 12). * was considered different from baseline. Data are expressed as means ± standard deviation.

Glucose concentrations were increased immediately after the race in the DEC (basal: 86.6 ± 16.69 vs. final: 113.0 ± 11.27 mg/dL, *p* < 0.0001) and CAF (basal: 81.92 ± 12.02 vs. final: 107.75 ± 13.08 mg/dL, *p* < 0.0001) groups, without differences between the trial groups (Figure 2A). Additionally, lactate concentrations were also enhanced immediately after the race in the DEC (basal: 0.78 + SD vs. final: 9.14 + 6.97 nmol/L, *p* = 0.0007) and CAF (basal: 1.28 + 0.88 vs. final: 9.34 + 6.07 nmol/L, *p* = 0.0004) groups, with no difference between trials (Figure 2B).

### 3.4. Blood Pressure Levels

**Figure 3:**
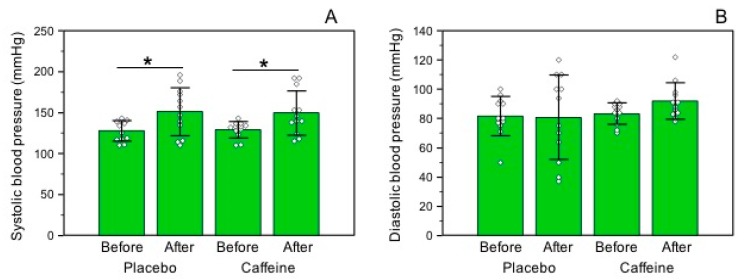
Blood pressure before and after the trials. (**A**) Systolic blood pressure levels (*n* = 12) and (**B**) diastolic blood pressure levels (*n* = 12). * was considered different from baseline. Data are expressed as means ± standard deviation.

Systolic blood pressure levels were increased immediately after the race in the DEC (basal: 127.83 + 12.43 vs. final: 151.3 + 29.24 mmHg, *p* = 0.002) and CAF (basal: 129.25 + 10.05 vs. final: 149.67 + 27.06 mmHg, *p* = 0.012) groups, without differences between the trials (Figure 3A). On the other hand, diastolic blood pressure levels did not change after the race with ingestion of DEC (basal: 81.58 + 13.44 vs. final: 80.83 + 28.79 mmHg, *p* = 0.459) and CAF (basal: 83.33 + 7.30 vs. final: 91.92 + 12.38 mmHg, *p* = 0.050), with no difference between the trials (Figure 3B).

### 3.5. Performance and Ratings of Perceived Exertion

**Figure 4:**
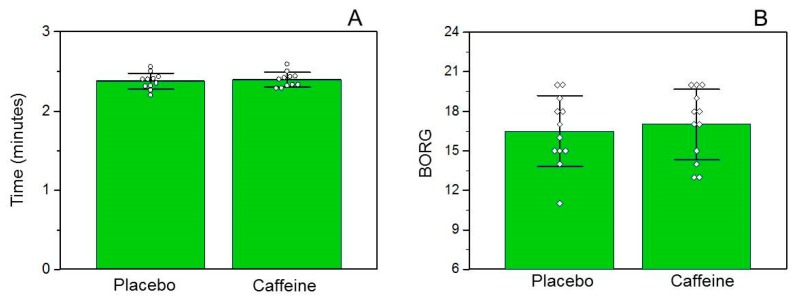
Time trial performance and ratings of perceived exertion (RPE) between trials. (**A**) Time trial performance (*n* = 11) and (**B**) BORG scale (*n* = 12). Data are expressed as means ± standard deviation.

Time trial performance was not enhanced between trials (DEF: 2.38 + 0.10 vs. CAF: 2.39 + 0.09 min, *p* = 0.336) (Figure 4A), nor was the RPE scale (DEC: 16.50 + 2.68 vs. CAF: 17.00 + 2.66, *p* = 0.326) (Figure 4B).

## 4. Discussion

The main finding of this study was that the ingestion of CAF did not improve the time trial performance of an 800-m run in overnight-fasting runners, when compared to DEC. In addition, RPE, blood pressure levels, and blood glucose and lactate concentrations did not change between the trials. The increase in systolic blood pressure levels, blood glucose and lactate concentrations after the race, were independent of caffeine; in other words, there was an exercise-induced physiological effect.

Despite the probable anaerobic contribution of caffeine supplementation in low carbohydrate availability conditions [8,14], in the present study the time trial performance was not enhanced. This could be explained by the large variations in coffees nutritional content, which can suffer changes in content of chlorogenic acid, caffeic acid, caffeine [6] due to climate conditions, coffee processing (roasting, storage, preparation), and genetic engineering manipulations [7,21]. Likewise, Graham et al. [4] state that coffee-derived chlorogenic acid may explain the absence of caffeine effects in performance, as chlorogenic acid could blunt the caffeine’s ergogenic aid. However, Hodgson et al. [17] observed that the presence of chlorogenic acid in CAF and DEC seems not to change the ergogenic effects of caffeine during cycling.

Moreover, evidence suggests that coffee is rich in theophylline, which might have caffeine-like actions, as it inhibits adenosine receptors and increases carbohydrate oxidation during a 30-min cycle ergometer exercise at 75% VO_2max_ [22]. Thus, it is plausible that either the CAF or DEC had a high quantity of theophylline, which resulted in a higher carbohydrate and energy supply to perform the run. However, to eliminate this doubt, inclusion of a third group (placebo) using just water could better explain these findings.

The discrepancies in studies involving coffee ingestion and running may be due to the consumption of different caffeine contents in coffee, which, as in the present study, was not quantified in the studies of Clarke et al. [3] and Wiles et al. [5]. It could also be due to different protocols performed to evaluate exercise performance. Although meta-analysis data suggests that ergogenic effects of caffeine may be assigned to endurance exercises; with particular focus on performance (time to exhaustion) protocols [4,16], no enhancement of time performance was observed when CAF was consumed one hour before the run until voluntary exhaustion, compared to DEC or the placebo. Jeukendrup et al. [23] suggest that exhaustion protocols are extremely variable from day to day (coefficient of variation of 27%). In the present study we evaluated performance using the time trial performance and no beneficial caffeine effects were found; however, using a similar protocol regarding performance, Clarke et al. [3] and Wiles et al. [5] found enhanced time trial performances. Therefore, future studies are needed to better understand which exercise performance protocol might be more appropriate to test ergogenic supplements.

It should be noted that the absence of positive caffeine effects may be related to polymorphisms in the CYP1A2 (metabolizes caffeine) and ADORA2A (adenosine receptor) genes [11]. Recently, a study showed that those with the AA genotype, who are faster metabolizers of caffeine than those with the CC and AC genotypes, benefited with better performances. For example, isolated caffeine powder (2 mg/kg b.w.) was able to reduce the time trial by 4.8% and 4 mg/kg b.w. by 6.8%. In contrast, those with the CC genotype did the time trial in a higher time of 13.7% after consuming 4 mg/kg b.w. of isolated caffeine powder; no difference was reported for the AC genotype [24].

In this present study we did not observe a reduction in RPE. Guest et al. [24] found that caffeine does not attenuate RPE in all cyclists, this effect may be altered by the genotype of CYP1A2. Wiles et al. [5] observed that 3 g (150–300 mg/caffeine) of CAF one hour before exercise enhanced performance in a 1500-m race when compared to DEC; however, no difference was detected in RPE.

We did not find a difference in blood lactate and glucose concentrations or blood pressure between trials; however, there was an enhancement due to an exercise-induced effect. Therefore, this increase may have been physiological by exercise and not supported by CAF.

The present study has some strengths. First, we evaluated participants who are frequent runners (4.9 times per week). Second, although no alteration in performance was related to habitual caffeine ingestion [10], we also identified the habitual quantity of caffeine ingested. In addition, we measured the main food sources containing caffeine and the frequency of consumption of caffeine-containing foods, a point few have reported through studies involving coffee use and running. Third, we evaluated the temperature and relative humidity, and no differences were observed. Likewise, no studies involving coffee ingestion and running [3,4,5] have evaluated the ambient temperature and relative humidity between trials, which may affect the performance. Fourth, we used CAF, a routine beverage; it seems interesting for clinical practice, as discussed by other authors [1].

A weakness of this study is that it included a very healthy group of participants with a very consistent schedule of physical activity; therefore, the amount of caffeine did not have any effect, probably due to their well-established physical activity schedule and healthy diet. In addition, the sample size was very small, so further studies with a larger number of participants are needed to explain the effects of caffeine’s ergogenic aid on performance. Moreover, we performed only two trials with coffee, another group with a placebo using just water could eliminate the effect of all runners thinking that they are consuming CAF, and the performance effect could be lost. Additionally, we did not evaluate the CYP1A2 and ADORA2A genotypes, which may be associated with different responses to caffeine-induced performance [11,24]. Although all runners were advised to maintain their normal hydration, we did not control to ensure participants were euhydrated before doing the exercise. While hydration was unlikely a factor in this short exercise bout, their status at the start could impact their performance. Therefore, further studies are encouraged to control this variable.

## 5. Conclusions

CAF consumption failed to improve the time trial performance of an 800-m run in overnight-fasting runners, when compared with DEC. Moreover, no difference was found in RPE, blood pressure levels, and blood glucose and lactate concentrations between the two trials.

## Figures and Tables

**Table 1:** General characteristics.

Variables	Mean ± SD
Age (years)	23.50 ± 3.94
Exercise frequency (times/week)	4.92 ± 1.62
Distance of race (km)	2.83 ± 2.25
Body weight (kg)	70.38 ± 8.41
Body mass index (kg/m^2^)	21.83 ± 3.43
Waist circumference (cm)	75.50 ± 3.99
Thigh circumference (cm)	52.46 ± 4.71
Calf circumference (cm)	34.75 ± 3.14
Triceps skinfold (mm)	17.17 ± 6.00
Subscapular skinfold (mm)	18.17 ± 4.93
Suprailiac skinfold (mm)	17.75 ± 7.07
Thigh skinfold (mm)	23.58 ± 6.91
Ambient temperature–decaffeinated group (°C)	21.33 ± 0.49
Ambient temperature–caffeinated group (°C)	21.42 ± 0.51
Ambient relative humidity–decaffeinated group (%)	89.00 ± 3.36
Ambient relative humidity–caffeinated group (%)	87.67 ± 4.16

**Table 2:** Food intake of runners.

Nutrients	Mean ± SD
Calories (kcal)	2340.56 ± 784.20
Carbohydrates (%)	45.81 ± 12.13
Carbohydrates (g/kg of b.w.)	4.02 ± 2.18
Protein (%)	23.51 ± 6.23
Protein (g/kg of b.w.)	1.92 ± 0.81
Lipids (%)	30.61 ± 9.26
Lipids (g/kg of b.w.)	1.09 ± 0.37

**Table 3:** Food frequency and quantity of caffeine from foods*.

Foods	Daily Frequency Mean ± SD	Daily Quantity Mean (Min–Max)	Caffeine Mean (Min–Max) (mg/d)
Caffeinated coffee (mL)	0.83 (0–3)	137.50 (0–600)	35.75 (0–156)
Chocolate (g)	0.33 ± 0.49	32.75 ± 56.42	6.55 (0–30)
Ready-to-drink chocolate (mL)	0.25 ± 0.45	66.66 ± 130.26	2.00 (0–12)
Cola soft drink (mL)	0.41 ± 0.90	150.00 ± 306.00	12.00 (0–80)
Caffeine powder (mg)	0.16 ± 0.38	35.00 ± 121.24	35.00 (0–420)
Total caffeine (mg/d)	-		91.30 (6–420)

* Caffeine content was acquired from USDA Food Composition Databases [20] or food labels.
